# Immunotherapy of Childhood Sarcomas

**DOI:** 10.3389/fonc.2015.00181

**Published:** 2015-08-07

**Authors:** Stephen S. Roberts, Alexander J. Chou, Nai-Kong V. Cheung

**Affiliations:** ^1^Department of Pediatrics, Memorial Sloan Kettering Cancer Center, New York, NY, USA

**Keywords:** pediatric sarcoma, immunotherapy of cancer, antibodies, monoclonal, CAR T cells, tumor vaccines, natural killer cells, osteosarcoma

## Abstract

Pediatric sarcomas are a heterogeneous group of malignant tumors of bone and soft tissue origin. Although more than 100 different histologic subtypes have been described, the majority of pediatric cases belong to the Ewing’s family of tumors, rhabdomyosarcoma and osteosarcoma. Most patients that present with localized stage are curable with surgery and/or chemotherapy; however, those with metastatic disease at diagnosis or those who experience a relapse continue to have a very poor prognosis. New therapies for these patients are urgently needed. Immunotherapy is an established treatment modality for both liquid and solid tumors, and in pediatrics, most notably for neuroblastoma and osteosarcoma. In the past, immunomodulatory agents such as interferon, interleukin-2, and liposomal-muramyl tripeptide phosphatidyl-ethanolamine have been tried, with some activity seen in subsets of patients; additionally, various cancer vaccines have been studied with possible benefit. Monoclonal antibody therapies against tumor antigens such as disialoganglioside GD2 or immune checkpoint targets such as CTLA-4 and PD-1 are being actively explored in pediatric sarcomas. Building on the success of adoptive T cell therapy for EBV-related lymphoma, strategies to redirect T cells using chimeric antigen receptors and bispecific antibodies are rapidly evolving with potential for the treatment of sarcomas. This review will focus on recent preclinical and clinical developments in targeted agents for pediatric sarcomas with emphasis on the immunobiology of immune checkpoints, immunoediting, tumor microenvironment, antibody engineering, cell engineering, and tumor vaccines. The future integration of antibody-based and cell-based therapies into an overall treatment strategy of sarcoma will be discussed.

## Introduction

Sarcomas are a heterogeneous group of malignant tumors arising from bone or soft tissues. More than 100 different subtypes of sarcoma have been described in adults and pediatrics; the majority of cases in children are rhabdomyosarcoma, Ewing’s family of tumors, osteosarcoma, and the non-rhabdomyosarcoma soft tissue sarcomas. Although these tumors are rare individually, as a group they account for 10–14% of all childhood cancers (1). While most patients who present with localized disease are highly curable with conventional therapies involving surgery and chemoradiotherapy, those who present with metastatic disease or who relapse post-therapy have an extremely poor prognosis, with little to no improvements in survival seen over the past 20 years. Furthermore, current therapies are highly toxic and associated with significant long-term morbidity in survivors; thus, new and effective therapies are urgently needed for these patients.

## History of Immunotherapy for Sarcomas

That the immune system might be involved in cancer control was first observed in sarcoma patients when Wilhelm Busch in Germany reported in 1866 on tumor regressions in sarcoma patients who developed erysipelas infections (2). Immunotherapy for the treatment of sarcomas can be traced back at least as far as 1891, when William Coley, a prominent bone surgeon at Memorial Hospital in New York (now Memorial Sloan Kettering Cancer Center), published his report on the use of what came to be known as “Coley’s Toxin” to treat a series of sarcomas of the bone (3, 4). He found that injections with streptococcus organisms (originally live bacteria, later a heat-killed concoction that also included *Serratia marcescens*) could induce remissions in some patients with otherwise inoperable sarcomas. Though use of his toxins was highly controversial and eventually fell out of favor, they are considered by many to be the precursors of today’s modern anti-cancer immunotherapy (5). Perhaps the best conceptualization of what has become modern immunotherapy came from Paul Ehrlich in the early 1900s with his description of the “magischen kugeln” – the “Magic bullet” – specific medicines fashioned to attack and kill only the diseased cell while sparing the surrounding normal tissues (6). The increased frequency of lymphoid malignancies in patients with immunodeficiencies also suggests that the immune system plays an important role in carcinogenesis (7). In addition, development of sarcomas has been well described in allograft transplant recipients, with a risk more than double that of non-immunosuppressed patients (8).

## Immune System in the Non-Malignant State

Our immune system is a complex organization of immune cells and mediators that interact with each other and with other accessory cells to protect against infections; simultaneously, this system must maintain tolerance toward self. The immune system consists of two layers of defense: the innate and adaptive spheres. The innate immune system includes dendritic cells, mast cells, and macrophages, as well as natural killer (NK) cells, neutrophils, basophils, and eosinophils. Innate immune cells serve as the initial defense against foreign antigens. Once activated, macrophages and mast cells release cytokines that engage additional immune cells and initiate an inflammatory response. Dendritic cells serve as antigen-presenting cells, taking in foreign antigens and subsequently presenting them for recognition by adaptive immune cells, thereby recruiting the second sphere of the immune system. NK cells can also interact with dendritic cells, either activating or eliminating them depending on context, thus they too can influence both the innate and adaptive immune systems.

The adaptive immune system includes B-lymphocytes, CD4+ T helper lymphocytes, and CD8+ cytotoxic T lymphocytes (CTLs). This arm of the immune system requires direct activation through antigen presentation by antigen-presenting cells. Upon antigen presentation and activation, antigen-specific T and B cells are generated. Together, the innate and adaptive pathways eliminate pathogens and remove damaged cells (7, 9). Unlike the innate system, the adaptive immune response requires training, but, once established, is antigen specific, has a memory, and can be recalled to rapid action in the future.

## Immune Surveillance and Immunoediting

One of the basic principles of cancer immunosurveillance is that cancer cells possess antigens that distinguish them [or set them apart] from non-transformed cells. These so-called tumor “neoantigens” can be recognized by the endogenous immune system and targeted for destruction. These tumor antigens are generally products of mutated genes, abnormally expressed normal genes, or genes coding for viral proteins. Unfortunately, transformed cells, under the selective pressure of the normal host response, are sometimes able to evolve evasive or immune-suppressive mechanisms and thus avoid detection and/or eradication. This concept that the immune system, while protecting against cancer, influences tumor immunogenicity and ultimately tumor escape was proposed as the framework for cancer immunoediting (10). This process can be divided into three phases: elimination, equilibrium, and escape. During the elimination phase both the innate and adaptive immune systems work to identify a developing neoplasm and eliminate it, through various mechanisms including activation of innate immune effector cells such as NK cells, and secretion of interferons (IFNs) and subsequent activation of dendritic cells, which in turn promote adaptive anti-tumor immune responses. However, a subset of cancer cells may develop the ability to survive this elimination phase, and thus the developing neoplasm enters the equilibrium phase. Here, the immune system prevents tumor escape, yet fails to eradicate it completely and thus participates in influencing the immunogenicity of these remaining cells. Finally, in the escape phase, those tumor cells that evolved the ability to evade the immune system during the equilibrium phase progressively proliferate and present as clinically apparent tumors. Mechanisms by which this escape may occur include loss of tumor antigens, down regulation of histocompatibility locus antigens (HLA) from the tumor cell surface; altered tumor microenvironment that is immunosuppressive due to the recruitment of regulatory T cells (Tregs); myeloid-derived suppressor cells, tumor-associated M2 macrophages, and others (11–13); upregulation of inhibitory receptors (e.g., PD-1) on T cells; or upregulation of inhibitory ligands (e.g., PD-L1 or B7-H3) on stromal cells or tumor cells.

## Immunomodulatory Agents

A variety of immunomodulatory agents have been investigated for the treatment of sarcomas, including cytokines such as interleukin-2 (IL-2) and IFN. The majority of sarcoma studies have been conducted in adult patients with advances fueling interest in the pediatric patient population.

### Cytokines

Stimulation of the immune system has been attempted using various cytokines. Cytokines are involved in a wide array of immune functions including modulation of antigen presentation and T cell activation (14, 15). The list of cytokines continues to expand (15). Although the most widely studied clinically are IFN and IL-2, several other cytokines are also moving into the clinic.

#### Interleukin 2

Interleukin 2 stimulates T cells proliferation, induces generation of CTLs, and facilitates the maintenance of NK cells (16–18). IL-2 is FDA approved for the treatment of metastatic renal cell carcinoma and melanoma, and responses to IL-2 have been reported in several other cancers including lung and breast cancers (19, 20). In pediatrics, IL-2 has been used most notably for the treatment of high-risk neuroblastoma in combination with an anti-GD2 monoclonal antibody (mAb) and granulocyte-macrophage colony-stimulating factor (GM-CSF) (21). A study of high-dose IL-2 in relapsed pediatric patients included four patients with osteosarcoma and two patients with Ewing sarcoma. Two of the four osteosarcoma patients had complete responses, while the other two and both Ewing sarcoma patients had progressive disease (20). However, use of high-dose infusional IL-2 is greatly hampered by significant toxicity, including capillary leak syndrome; continued use in pediatric sarcoma as a single agent seems unlikely. Several studies are ongoing in pediatrics combining IL-2 given in a variety of different routes and dosages with antibody therapy, vaccines, and adoptive cell therapy.

#### Interferon

Interferons are a complex family of molecules that bind to IFN receptors; IFNα and IFNβ activate type I receptors, while IFNγ activates type II receptors (15, 22). Both IFNα and IFNβ activate immune cells and increase antigen presentation to T cells. INFα is approved for use in melanoma and has also been studied in sarcomas. Most recently, the large EURAMOS study reported three-year follow-up data on 715 pediatric and adult osteosarcoma patients up to 40 years of age randomized to postoperative chemotherapy ± IFN; there was no survival benefit from IFNα when added to standard three-drug chemotherapy in osteosarcoma patients (74% chemotherapy alone vs. 77% chemotherapy + IFNα; EFS, *p* = 0.21) (23). Further development of IFN as a single agent in pediatric sarcoma seems unlikely; its role in pediatric sarcoma immunotherapy as an adjuvant combined with other immunotherapies such as adoptive cell therapy to increase antigen presentation remains to be defined.

#### Interleukin 15

Interleukin 15 (IL-15) (24, 25) is a 14–15 kDa glycoprotein that binds to a heterotrimeric receptor that shares the IL-2R/IL-15Rβ (CD122) and the common gamma (γc) chain (CD132) with the IL-2 receptor (26), as well as a unique α subunit (IL-15Rα) that confers receptor specificity. However, unlike IL-2, IL-15 is not required for the maintenance of Tregs (27); it does not induce activation-induced cell death (AICD) of CD8+ effector T cells (28); is required for the differentiation of NK, effector CD8+ and memory phenotype CD8+ T cells; and does not cause capillary leak syndrome (29). IL-15Rα binds to IL-15 with high affinity (Kd < 10^–11^ M) and retains IL-15 on the cell surface. IL-15Rα trans-presents IL-15 to IL-2R/IL-15Rβ-γc on neighboring NK and T cells through immunological synapses (30, 31). IL-15 has diverse immunologic effects (26). It stimulates the proliferation of activated CD4−CD8−, CD4+CD8+) CD4+, CD8+ T cells, induces cytotoxic CTLs, and stimulates the generation, proliferation, and activation of NK cells. Though not essential for the generation of memory CD8+ T cells, IL-15 is required for their homeostatic proliferation over long periods of time (32). IL-15 protects neutrophils from apoptosis, modulates phagocytosis, stimulates mast cell growth, induces B cell proliferation and differentiation partially independent of T cell help, and increases their immunoglobulin secretion, while stimulating secondary cytokine release from macrophages and maturing dendritic cells. When given as the IL15/IL15Rα complex, it is more effective and should be less toxic than the soluble IL15 (33–35). Several preclinical studies have shown that IL-15 may potentiate anti-sarcoma immunotherapy in Ewing and osteosarcoma models (36–38). A clinical trial combining recombinant human IL15 with NK cells for relapsed and refractory pediatric solid tumors, including sarcomas, is currently underway at the U.S. National Cancer Institute (NCT01875601). Although no clinical trial of IL-15 has been conducted specifically for sarcomas, this cytokine will likely play a major role in future immunotherapy strategies.

### Liposomal-muramyl tripeptide phosphatidyl-ethanolamine

The immune modulator liposomal-muramyl tripeptide phosphatidyl-ethanolamine (L-MTP or mifamurtide) has been extensively studied, primarily in osteosarcoma. This compound is a non-specific modulator of innate immunity and is a synthetic analog of muramyl dipeptide derived from bacterial cell walls. It activates monocytes and macrophages leading to an increase of a wide variety of immunomodulatory molecules including: tumor necrosis factor-alpha (TNF-a), interleukin (IL)-1, IL-6, IL-8, IL-12, nitric oxide, prostaglandin E2, lymphocyte function-associated antigen 1 (LFA-1), and intercellular adhesion molecule 1 (ICAM1) (39). Preclinical studies suggested that this inflammatory response triggered by L-MTP could potentially eliminate minimal residual disease. A small study conducted by the EORTC Soft Tissue and Bone Sarcoma Group in the 1990s treated 20 adult patients with soft tissue sarcomas with MTP; there were no responses in that study (40). The largest clinical experience with combination chemotherapy and L-MTP derives from the Intergroup (INT-) 0133 osteosarcoma study. This prospective, double randomization, phase III trial tested first the utility of adding ifosfamide to the standard three-drug chemotherapy regimen (doxorubicin, cisplatin, and high-dose methotrexate); and second the impact on survival with the addition of L-MTP to either assigned chemotherapy arm. No difference in survival was found for patients who received ifosfamide in addition to the standard three-drug chemotherapy. The study did suggest that L-MTP had a beneficial impact on survival, improving the 5-year overall survival rate from 70 to 78% (*p* = 0.03) (41). However, when the 91 patients who had metastatic disease were analyzed separately, the difference in survival between those who did versus those who did not receive L-MTP, though suggesting improvement, did not reach statistical significance. The overall survival at 5 years was 53% for those randomized to receive L-MTP versus 40% for those who did not (*p* = 0.27) (42). Based, in part, on the updated results of the non-metastatic cohort of INT-0133, the European Medicines Agency granted L-MTP an indication for the treatment of non-metastatic osteosarcoma in 2009; the American Food and Drug Administration (FDA) did not. L-MTP is also approved for use in Turkey, Mexico, and Israel.

## Antibody-Based Immunotherapy

### Monoclonal antibodies

Unmodified antibodies specific for tumor-associated surface antigens can engage tumor cells while activating innate immune effector cells, primarily macrophages and NK cells via their Fc receptors (FcγR). Once activated, the effector cell releases cytotoxic granules to kill the target cell, a process known as antibody-dependent cellular cytotoxicity (ADCC). It is important to note that T cells do not possess FcγR and have no affinity for conventional antibodies, and hence cannot be activated by these tumor selective antibodies.

Many mAbs have been developed for various cancer types. While there have been notable successes (for example, anti-CD20 for hematologic malignancies, anti-human epidermal growth factor receptor 2 (HER2) for breast cancer, and anti-GD2 for neuroblastoma), most mAbs have failed to improve outcomes despite their initial promise, especially in pediatric sarcomas.

Approximately 50% of osteosarcomas overexpress HER2, and HER2 expression was shown to correlate with a poorer prognosis (43); a phase II study was conducted by the Children’s Oncology Group (COG) to evaluate if the addition of trastuzumab (anti-HER2, Herceptin) to standard chemotherapy would improve survival in metastatic osteosarcoma patients. Ninety-six patients were enrolled, and 41 were found to have HER2 overexpression. Unfortunately, no significant difference in survival was seen in patients who received trastuzumab + chemotherapy compared to those who received chemotherapy alone {EFS of 32% in both arms, OS of 50% for chemotherapy alone compared to 59% for chemotherapy + trastuzumab, [*p* = 0.54 for EFS; *p* = 0.58 for OS] (44)}.

Instead of binding directly to tumors, antibodies can neutralize growth factors (e.g., insulin-like growth factor 1 (IGF1) or IGF2) or their receptors (e.g., IGF-1R, -A12). A large body of preclinical and early clinical data suggested that IGF1 and 2 might play an important role in the initiation and progression of a variety of cancers, including pediatric sarcomas (45–47). Several phase I and II studies were conducted evaluating anti-IGF1 mAbs in relapsed and refractory solid tumors including sarcomas, the largest being a phase II study by the COG that enrolled 116 patients, including 20 with rhabdomyosarcoma, 11 with osteosarcoma, and 10 with synovial sarcoma; there were no objective responses in any of the sarcoma patients (48, 49). Finally, a randomized phase II study of standard chemotherapy ± the anti-IGF-1R mAb ganitumab is ongoing within COG for Ewing sarcoma (NCT02306161). However, this agent failed to show improved outcomes in a large randomized phase III trial of adult pancreatic cancer patients (50) and the manufacturer has announced that they will not be pursuing development of this agent. Thus, regardless of the results of the ongoing trial, its future for pediatric sarcoma is unclear.

Although both IGF-1 and IGF-2 activate IGF-1R, the latter shares a similar tetrameric α2β2 structure with insulin receptor (IR). The IR can be expressed in two isoforms (IR-A and IR-B). IR-A binds to IGF-2 with the same affinity as it binds to insulin. In addition, insulin and IGF-1 receptor subunits can form hybrid heterodimeric receptors (51). Antibodies against IGF-1R only partially inhibit IR-A activity by disrupting the IR-A/IGF-1R hybrid, but completely fail to inhibit IR-A homodimers. Failure of IGF-1R inhibition results from two compensatory mechanisms: (1) IGF-2 is increased during treatment with IGF-1R mAb (52) which signals through IR-A, which is known to promote cancer survival (53). (2) Compensatory activation of the epidermal growth factor receptor (EGFR) allowing the cancer to continue to progress despite blockade of the IGF pathway (54). One novel approach to overcome these limitations is to reduce the serum and tissue levels of the IGF ligands, using neutralizing mAbs specific for both IGF-1 and IGF-2. By removing IGF-2, the escape mechanism of IGF-2-mediated IR-A activation can be aborted, suggesting that newer mAbs that target both IGF-1 and IGF-2 may have more success than the first-generation mAbs tested (55, 56).

Several trials of mAbs against the EGFR and the VEGFR (57) alone and in combination with chemotherapy have been conducted in children and young adults with sarcomas. The COG conducted a randomized trial of bevacizumab (anti-VEGFR) combined with vincristine, topotecan and cyclophosphamide in patients with recurrent Ewing sarcoma, as well as a randomized trial of bevacizumab and temsirolimus in combination with vinorelbine and cyclophosphamide in recurrent/refractory rhabdomyosarcoma patients. In the rhabdomyosarcoma trial, the bevacizumab arm was significantly worse than the temsirolimus arm and the study was stopped early (58); results for the Ewing sarcoma trial have not yet been published. Despite preclinical rationale for these targets (59–61), overall, these studies have not shown many significant responses in sarcomas, though some studies are ongoing.

A phase I trial of the anti-tumor necrosis factor-related apoptosis-inducing ligand receptor 2 (TRAIL-2) mAb lexatumumab was conducted by the U.S. National Cancer Institute (NCI) in pediatric solid tumors. This study enrolled 24 patients, including 21 with various sarcomas. No objective responses were seen and this mAb is no longer under clinical development (62).

Given the success of anti-GD2 mAb therapy in neuroblastoma (21, 63) and the expression of GD2 by many sarcomas (64, 65), studies exploring the use of these mAbs in sarcomas, particularly in osteosarcoma, are underway. Current trials include the anti-GD2 mAbs humanized3F8 (NCT01419834 and NCT01662804) and hu14.18K322A (NCT00743496).

### Engineered antibodies including bispecific antibodies

**Figure 1:**
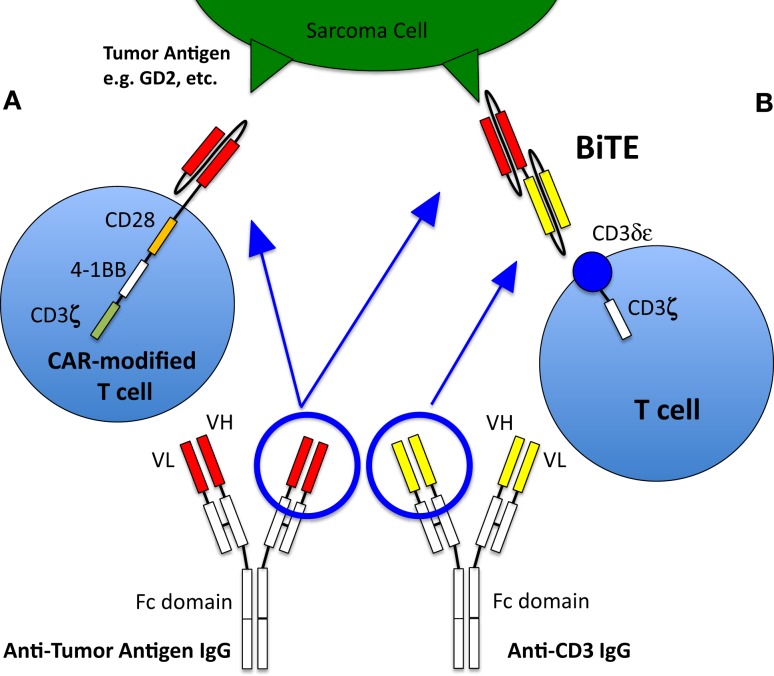
**T cell activation and recruitment to tumor cells**. Normally, T cells will only target cells via antigenic sequences presented to the T cell receptor via MHC. However, this rarely occurs, especially in pediatric sarcomas, thus native T cells generally are not active in killing tumor cells and other strategies to recruit T cells to the tumor are therefore required. **(A)** T cell with a chimeric antigen receptor to a sarcoma tumor-associated antigen is able to recognize the antigen and activate the T cell, allowing it to kill the targeted cell. The CAR-T cell depicted here is a so-called third generation CAR, because it contains three co-stimulatory domains (CD28, 4-1BB, and CD3ζ). **(B)** Bispecific antibody binding to a sarcoma tumor-associated antigen as well as to the CD3 receptor of a T cell, thus activating the T cell and allowing tumor cell killing. Figure adapted from Suzuki et al. (67).

Bispecific antibodies are engineered antibodies linking a tumor antigen recognition domain to a second domain that activates a receptor on immune effector cells, typically T cells (Figure 1). The anti-CD19/anti-CD3 bispecific antibody blinatumomab was approved by the FDA for the treatment of precursor B cell acute lymphoblastic leukemia in 2014, making it the first in its class to be approved in the US. Recently published preclinical data of an anti-GD2 T cell retargeting bispecific antibody showed excellent *in vivo* activity against GD2 expressing neuroblastomas and melanomas (66). Currently, there are limited clinical data on bispecific antibodies in pediatric sarcomas; there is one study that recently began enrolling OS patients (Activated T Cells Armed with GD2 Bispecific Antibody in Children and Young Adults With Neuroblastoma and Osteosarcoma, NCT02173093).

### Immunologic checkpoint blockade or inhibitors

Recently, there has been much excitement about the potential of the immune checkpoint inhibitors in solid tumors including pediatric sarcomas following their clinical successes and approvals for treatment of metastatic melanoma and metastatic squamous non-small cell lung cancer.

### CTLA-4 blockade

Ipilimumab is a human IgG4 monoclonal antibody that blocks the anti-cytotoxic T-lymphocyte-associated protein 4 (CTLA-4) and was the first of the new generation of checkpoint inhibitors to gain FDA approval (68). CTLA-4 is a member of the immunoglobulin superfamily; after T cell activation, CTLA-4 is expressed on the plasma membrane of cells where it acts to inhibit T cell function through a variety of mechanisms, allowing tumor cells to escape immune surveillance (69, 70). The experience of ipilimumab in pediatric patients is limited; GI toxicity was the major concern. A small phase II study in adults with synovial sarcoma had no clinical or immunological responses (71).

### PD-1 blockade

Antibodies targeting the programed cell death protein 1 pathway (PD-1/PD-L1) (nivolumab, pembrolizumab) function in a similar manner to ipilimumab by removing the brakes on T cells which then can perform active anti-tumor immune surveillance (69, 70). Preclinical studies have demonstrated expression of PD-1L in OS and suggest that high expression levels may correlate with worse clinical outcomes (72); *In vivo* studies using murine sarcoma models with anti-CTLA-4 antibodies have also shown promise for these agents (73). Currently, however, these agents have limited pediatric clinical data available; several trials with these agents for relapsed or refractory pediatric solid tumors are currently ongoing.

Despite the overall successes of checkpoint inhibitors, only subsets of patients with melanoma, lung cancer, ovarian cancer, NHL, and Hodgkin lymphoma have responded. Two important studies have examined the tumors of responders versus non-responders, one in melanoma and one in non-small-cell lung cancer (74–76). In both cases, treatment efficacy was associated with a higher number of mutations in the tumors. In melanoma patients treated with ipilimumab, the investigators carefully examined the tumors of those who responded versus those who did not, and found that the responders had tumors with higher mutation rates and tumor antigens and in particular, those whose tumor neoantigens shared tetrapeptide sequences with viral antigens were most likely to be responders to checkpoint inhibition (75). To improve on the quality of response to immune checkpoint blockade, CTLA-4 and PD-1/PD-L1 antibodies are being tested in combination or when added to other anti-cancer agents such as chemotherapy, targeted therapy, radiotherapy, and other immunotherapy (19, 69, 77). Currently, the COG is conducting a phase I/II study (NCT02304458) of nivolimab alone or in combination with ipilimumab for relapsed and refractory solid tumors including sarcomas.

Although there is much excitement currently surrounding these new agents, caution seems warranted in pediatric sarcomas. In contrast to melanoma and lung cancer, pediatric cancers in general and pediatric sarcomas in particular have an extremely low rate of recurrent mutations (<1 mutation per Mb for pediatric cancers compared to 15 per Mb for melanomas) (78, 79). Furthermore, many sarcomas do not express major histocompatibility complex (MHC) which is required for both the afferent and efferent arms of T cell response (80). Taken together, it seems probable that checkpoint inhibitors may have less efficacy in pediatric sarcomas (especially as single agents) than in melanoma and lung cancer; careful consideration of ideal clinical trial design using these agents will be critical for defining their potential role in the immunotherapy of pediatric sarcomas.

## Tumor Vaccines

Vaccines directed against specific tumor antigens were some of the earliest targeted immunotherapies tested. The aim of tumor vaccines is to induce an anti-tumor response through exposure to tumor antigens. Of the high value tumor targets among the 75 candidates derived from the NCI consensus panel, only a few are directly adaptable to sarcoma (81). The most notable are the gangliosides GD2 and GD3, polysialic acid, and translocation breakpoints. In animal models and human clinical trials (9, 82), vaccines have shown efficacy in preventing tumor development or delaying progression, but have generally failed to mediate regressions of established tumors. Single-arm trials have investigated vaccines targeting whole cells, lysates, proteins, and peptides in both adult and pediatric patients with sarcomas. Results from most studies in sarcomas (adult and pediatric) have been disappointing, though some have shown potential benefit with either laboratory evidence of the development of an immune response, or prolonged stable disease or disease-free intervals (83–88). Several additional pediatric sarcoma studies remain ongoing (NCT01241162, NCT01803152, NCT01061840). Promising results of a recent phase I study of a bivalent GD2-GD3 gangliosides vaccine in combination with β-glucan in neuroblastoma patients in second remission (89) suggest that vaccines for sarcomas may be beneficial if given in the setting of minimal residual disease.

## Adoptive Cell Therapy

Adoptive cell therapy is the term coined to describe the concept of giving a patient immune cells with cytolytic properties in sufficient numbers to cause an anti-tumor response. There are various strategies to accomplish this, including use of *ex vivo* expanded autologous cells and infusion of donor-derived allogeneic immune effector cells.

### Natural killer cells

Natural Killer cells are lymphocytes of the innate immune system with both cytotoxic and regulatory functions and are important mediators of immune responses against infections and cancer. Unlike T and B cells, NK cells recognize their targets without prior sensitization and generally do not have the same memory system [with some exceptions (90)] as T or B cells. NK cells are activated through various receptors that recognize proteins that are upregulated by cell stress or are foreign. In turn, NK cells are negatively regulated by inhibitory receptors that primarily bind HLA as a means of preventing self-recognition, thus preventing autoimmunity. NK cell target cytotoxicity is triggered when the overall balance between the various activating and inhibitory signals is weighted toward activation (91). NK cells were initially identified through their ability to kill tumor cells (92); the anti-tumor actions of NK cells have subsequently been documented in many human and animal models. NK cells are neither HLA-restricted nor do they require activation via the adaptive immune system (93). These facts plus their ability to target and kill a wide variety of tumors has led to strong interest in their therapeutic potential (94). The first application of NK-cell-enriched cellular products to treat cancer was performed at the NCI using autologous cells in 1980 (95). Subsequently, clinical trials, primarily in acute myeloid leukemia, confirmed that haploidentical donor-derived NK cells can be expanded *in vivo* and can induce remissions (96). Preclinical data suggest that NK cell strategies may be of benefit in pediatric sarcomas (97). Specifically, various studies have shown that Ewing sarcoma, osteosarcoma, and rhabdomyosarcoma cell lines, including highly chemoresistant lines, are all sensitive to NK cell killing and that cytokine activation greatly enhances this killing ability both *in vitro* and in *in vivo* models (36, 38, 98). Several current NK-cell-based studies are open to pediatric sarcomas and apply various strategies, including post-allogeneic transplant and *ex vivo* expansion and/or cytokine stimulation; however, no results have yet been reported from these trials.

### Cytotoxic T lymphocytes

Cytotoxic T Lymphocytes are highly efficient at targeting and killing specific cells; thus, there has long been much interest in harnessing this ability for cancer immunotherapy. *De novo* T cells are generally of low frequency and incapacitated by the tumor microenvironment. Initial efforts to use T cells for cancer therapy involved *ex vivo* expansion of the so-called tumor infiltrating lymphocytes (TILs) freed from excised tumors. This approach is limited, however, by the fact that they cannot be reliably extracted or be expanded to sufficient numbers from most tumors. To date, there are no studies in pediatric cancer patients (9). Despite their limitations, TILs are an important proof-of-concept of the potential value of T-cell-based immunotherapy as they were the first immunotherapy to induce regressions of bulky tumors (99). To overcome these limitations, polyclonal T cells can be genetically modified to express T cell receptors (TCRs) that recognize tumor peptide antigens in the context of MHC. These transgenic TCRs function like their natural counterparts, but remain restricted by MHC, thus limiting the use of these cells to the patient’s specific individual HLA alleles. As approximately 50% of the Caucasian population in the U.S. express HLA A*0201, many studies have focused on associated antigens, particularly the cancer testis antigens. Among these, NY-ESO-1 is one of the most studied with expression found in 70–80% of synovial sarcomas, but only sporadically in other sarcomas (100, 101); in a pilot feasibility study, four of six patients with synovial sarcoma had an objective response (101, 102). Further studies using NY-ESO-1 CTLs in synovial sarcoma are ongoing (NCT01343043).

### Chimeric antigen receptor-modified T cells

Because T cells do not carry Fcγ-receptors, these potent effector cells cannot recognize tumor-bound antibodies, and have therefore traditionally not been recruited by such antibodies to tumor sites. Furthermore, T cells need to recognize tumor peptides in the context of their own MHC antigens to be effective killers. However, many tumors down regulate or lose their HLA, or even tumor peptides, making them transparent to even the primed T cells. To overcome these issues of HLA and to broaden the selection of targets (e.g., to carbohydrates or lipids), chimeric antigen receptors (CARs) can be engineered into T cells. These receptors are not classic TCRs, but derived from conventional antibodies specific for any target. A chimeric molecule consisting of an antibody in the form of single chain Fv (scFv) as the ectodomain, and T cell signaling machinery as the intracellular domain, forms this artificial receptor through which T cells are activated when they come into contact with the specific antigen, without the necessity of MHC. These CARs are inserted into T cells using viral vectors, DNA transposons, or RNA transfection. In the early versions (so-called First generation) of CAR-modified T cells (CAR T cells), signaling was done through a single activation domain (either the CD3-ζ chain or FcϵRIγ). Second- and third-generation CAR T cells contain one or two additional co-stimulatory signaling domains such as CD28, 4-1BB, and OX40 (67) (Figure 1). The first-generation CAR T cells did not show significant activity in clinical trials presumably because many tumor cells lack co-stimulatory ligands (103), and because of poor persistence of the T cells, although a phase I study of anti-GD2 CAR T cells in relapsed neuroblastoma patients saw some objective clinical responses including complete remission in three patients (104, 105). Second-generation CAR T cells have shown improvements in T cell proliferation and survival (106) and have shown promising results in hematologic malignancies (107). Several studies with CAR T cells are underway that include pediatric sarcoma patients. Two of the open trials target HER2 expressing sarcomas (NCT00902044, NCT00889954), while two more target GD2 expression (NCT01953900, NCT02107963); it remains to be seen whether similar successes seen in hematologic malignancies can be achieved in solid tumors. The death of a patient receiving third-generation anti-HER2 CAR T cells has raised concerns regarding the safety of highly activatable T cells even when the expression of the antigen in normal tissues was low (NCT00924287) (108).

## Challenges

### Toxicity

In general, although immunotherapy may have less long-term toxicity than chemotherapy or radiation therapy, which is particular appealing for pediatric cancer, major short-term toxicities can be daunting. These include immediate infusion-related allergic reactions with mAbs, and autoimmune reactions to the checkpoint inhibitors, some of which were life threatening (109). In a recently completed phase I study of ipilimumab (NCT01445379) in pediatric patients with refractory solid tumors including sarcomas, no objective responses were seen but significant autoimmune toxicity was observed, with up to 50% of patients experiencing symptoms (Personal communication, Dr. L. Wexler, 2015); however, no pediatric safety data for these agents are yet published. In adults, enterocolitis, hepatitis, and dermatitis were the most commonly seen toxicities, but autoimmune-related toxicities due to unregulated T cell activity have been reported in nearly every organ system (109). Adoptive cell transfer also carries the real potential for serious adverse events. T cell therapy is highly potent such that even normal tissues with low target antigen expression can become innocent bystanders. These unintended and unexpected toxicities to critical organs can be life threatening (110) and have limited the choice of certain targets for redirected T-cell-based therapy (111). Additionally, T cells have been associated with severe, sometimes fatal, cytokine release. Cytokine release syndrome (CRS) occurs when extremely high levels of immune cells are activated thereby stimulating release of large amounts of inflammatory cytokines, leading to organ dysfunction and death. CRS is particularly seen with second- and third-generation CARs as well as bispecific antibodies (112), but can occur after antibody infusion as well as with other adoptive lymphocyte therapies. Corticosteroids are the mainstay of treatment, while anti-IL6R antibody can also be helpful (113).

### Target selection

Perhaps the most critical first step in designing cancer immunotherapy is identifying appropriate immunologic targets. A good immunotherapy target must be highly expressed on tumor tissues but not on normal tissues. Ideally, a good target will play a role in the underlying oncogenesis of the tumor, though this is not always required. Targets that meet these attributes are rare (81). An alternative approach has been to target markers that are highly expressed on cancers and expressed in the so-called non-vital tissues, such that targeting and loss of these normal cells are tolerated by the patient. Monoclonal antibody targeting of CD20, and CAR T cells and bispecific antibodies targeting CD19 are examples of this approach. Adding further difficulty to target selection is that they by necessity must be present on the surface of the cell for immune recognition, which limits the potential target list. In fact, of the 75 NCI consensus high value targets, two-thirds are internal antigens (81). The only way to target these internal antigens is through their peptides presented on the HLA; hence the description of such antibodies as TCR like. Less than 100 publications have been published on the discovery of such antibodies, but the best characterized are those against the RMFPNAPYL peptide of the Wilm’s tumor-1 (WT1) antigen presented on HLA0201 (114). However, this approach is limited by the restriction to specific HLA subtypes. Most pediatric sarcomas lack HLA expression (80), and among those that have it, only individuals with the specific restricted subtype would be sensitive to the immunotherapy. Efforts to mine gene expression databases for potential new antibody targets are promising but still in early stages; validation of these mRNA level exploratory analyses at the protein level will be critical (115, 116). Tables 1 and 2 list some of the pediatric sarcoma-specific targets, both MHC non-restricted (Table 1), and MHC restricted (Table 2), currently in preclinical and/or clinical development.

**Table 1:** **Cell surface targets for MHC non-restricted immunotherapy of pediatric sarcomas**.

Target	Tumor Expression	Normal Expression	Comments
GD2	Osteosarcoma (90%)	GD2+ neuronal tissue (peripheral sensory nerves)	Dinatuximab (Ch14.18) FDA approved for NB; trials in OS using hu3F8 and dinatuximab are planned.
	Soft tissue sarcomas (varies)		
HER2	Osteosarcoma DSRT	Low-level lung expression	
FGFR4	Rhabdomyosarcoma	Expressed during muscle development	
Glypican-3, -5	Rhabdomyosarcoma	Rare outside embryonal tissues	
FOLR1	Osteosarcoma, Rhabdomyosarcoma	Luminal cell mem-brane of some epithelial tissues	

*Table adapted from Orentas et al. (116)*.

**Table 2:** **MHC-restricted immunotherapy targets for pediatric sarcomas**.

Target	Tumor expression	Comments	Reference
NY-ESO-1	Synovial Sarcoma (70%)	Cancer testis antigen, HLA-A1	(102, 117)
HER2/Neu	Osteosarcoma (60%)		(64)
STEAP (Six-transmembrane epithelial antigen of prostate)	Ewing Sarcoma	% expression data limited	(118, 119)
WT1	Rhabdomyosarcoma (100%)	HLA-A1, A24, DP5, DR4	(120, 121)
	Ewing sarcoma (50%)		
PAX3-FKHR	Alveolar rhabdomyosarcoma (90%)	HLA-B7	(122)
SYT-SSX1, 2	Synovial Sarcoma (100%)	HLA-B7	(123)

*Table adapted from Orentas et al. (116)*.

## Future Directions

Sarcoma immunotherapy remains in its infancy. To date, while we have not seen the successes seen in other malignancies, there are glimpses of activity which suggest that immunotherapy could be an effective treatment modality. However, to fully realize that potential we believe that the following four areas must be carefully considered:

### Target discovery and validation

Given the narrow mutation landscape in sarcomas, and especially so among those with translocations, neoantigens derived from gene mutations are predicted to be rare. Translocation fusion sequences have remained difficult to target with T cells, or to be used as vaccines. Without neoantigens, even checkpoint blockades used at recommended dosage levels might not be effective. By default, differentiation antigens and tissue antigens deserve to be more carefully explored. These include the gangliosides GD2 (124) and GD3 (65), ROR2 (125), HER2 (126, 127), B7-H3 (128), CSPG4 (129, 130), polysialic acid (131), and glypican 3 (132). All of these antigens have established antibodies ready for construction of CAR T cells or bispecific antibodies. Importantly, most of these antibodies have already been tested in humans with acceptable toxicities. Considerations should also be given to novel engineered forms such as bispecific antibodies to retarget T cells (66) or bispecific antibodies for multistep targeting to greatly improve therapeutic index (133). Given the early glimpses of response to IGF1R antibodies and a better understanding as to why tumors escape, the new generation of dual-specific antibodies for IGF1 and IGF2 should be considered (56, 134).

### Careful patient selection

The majority of clinical trials to date have shown that immunotherapy is generally not effective against large, bulky disease. Thus, it is imperative that the proper patient population is selected for clinical trials moving forward. For example, we are developing a phase II anti-GD2 immunotherapy protocol for osteosarcoma patients in second or greater remission, with the goal of targeting pulmonary minimal residual disease. This is based on our experience in OS patients treated on our phase I protocol where we found that patients with visible metastatic lesions progressed rapidly while those with minimal residual disease have shown increased time to progression compared to historical controls. It would appear that the clinical efficacy of immunotherapy for pediatric sarcoma can best be tested in clinical trials designed to treat patients after their overt disease burden has been reduced as much as possible.

### Development of combined modality regimens

To date, the majority of studies using single immunotherapy modalities have not demonstrated significant activity in solid tumors in general and in pediatric sarcomas in particular. However, rational combinations of new immunotherapies are being developed and will need to be carefully explored. Antibodies combined with immunomodulatory agents are the most mature of these combinatorial approaches. Anti-GD2 mAbs combined with GM-CSF or GM-CSF and IL-2 are effective against neuroblastoma (21, 63) with studies planned in osteosarcoma. While checkpoint inhibitors, for reasons described above, are unlikely to be of significant benefit when used alone in pediatric sarcomas, their combination with adoptive cell therapy or bispecific antibodies has the potential to enhance the efficacy of these T cell-based strategies. Additionally, preclinical studies suggest that prior radiotherapy can induce tumor neoantigen expression and increased effectiveness of checkpoint blockade, echoing the abscopal effect in the clinic (135). Studies exploring this strategy in adults are underway and may be warranted in children. T cells could also be combined with NK cells: MHC down regulation by the tumor cells as a means of escape from T cell killing should render these cells more susceptible to NK cell killing, which does not require MHC, but is instead inhibited by high MHC ­expression (91).

### Tolerance of increased toxicity

This last point is perhaps the most controversial. However, historical precedent suggests that learning to manage toxicities associated with therapies can allow otherwise effective treatments to be developed. Anti-GD2 immunotherapy is associated with significant infusional toxicities including severe pain; this pain side effect was completely unexpected when these mAbs were first used (136). Fortunately, rather than halting the development of these antibody treatments, ways to overcome the toxicities were developed and as a result, anti-GD2 immunotherapy is now proven effective in neuroblastoma and is in active trials in sarcoma patients. Similarly, it seems likely that newer immunotherapy treatments, especially combination therapies as suggested above, will have both predictable, as well as unexpected, and potentially severe side effects. However, an unwillingness to carefully explore and manage novel toxicities may limit the adoption of some potentially beneficial treatments. With checkpoint blockade, the autoimmune toxicity seen shows that children do, in fact, have autoreactive T cells that will react with self if the “brakes” are sufficiently released. Since many tumors overexpress normal self antigens, it is plausible that “releasing the brakes” enough (by combining ipilimumab with nivolumab while pushing the dose of both) could allow an autoreactive T cell to target a protein on the tumor that would otherwise be tolerated by the immune system. The currently approved dose of ipilimumab for patients with melanoma, however, achieves the target trough concentration of 20 mcg/mL, the level at which ipilimumab attains maximum CTLA-4 blockade, in only 30% of patients (68), suggesting that increasing the dose could yield improved clinical benefit, if toxicities can be managed. Several clinical trials testing this hypothesis in adults are underway. Similarly, combination therapy with adoptive T cells and checkpoint blockade could have significantly increased toxicity, especially for on-target, off-tumor effects, such that appropriate target selection and clinical trial design to minimize these risks are critical.

## Conclusion

Pediatric cancer immunotherapy continues to advance; we believe these advances will improve outcomes in patients who have not benefited from conventional therapy alone. Late toxicities remain a major challenge for those patients who underwent life saving chemotherapy and radiation therapy. Immunotherapy offers an opportunity to consolidate remission while reducing genotoxic therapy. We are cautiously optimistic that immunotherapy will improve not just survival but also the quality of life in children with sarcomas.

## Conflict of Interest Statement

The authors declare that the research was conducted in the absence of any commercial or financial relationships that could be construed as a potential conflict of interest.
